# Prevalence of simian malaria parasites in macaques of Singapore

**DOI:** 10.1371/journal.pntd.0009110

**Published:** 2021-01-25

**Authors:** Meizhi Irene Li, Diyar Mailepessov, Indra Vythilingam, Vernon Lee, Patrick Lam, Lee Ching Ng, Cheong Huat Tan

**Affiliations:** 1 Environmental Health Institute, National Environment Agency, Singapore; 2 Parasitology Department, Faculty of Medicine, University of Malaya, Kuala Lumpur, Malaysia; 3 Saw Swee Hock School of Public Health, National University of Singapore, Singapore; 4 Biodefence Centre, Force Medical Protection Command, Headquarters Medical Corps, Singapore Armed Forces, Singapore; 5 School of Biological Sciences, Nanyang Technological University, Singapore; Temple University, UNITED STATES

## Abstract

*Plasmodium knowlesi* is a simian malaria parasite currently recognized as the fifth causative agent of human malaria. Recently, naturally acquired *P*. *cynomolgi* infection in humans was also detected in Southeast Asia. The main reservoir of both parasites is the long-tailed and pig-tailed macaques, which are indigenous in this region. Due to increased urbanization and changes in land use, there has been greater proximity and interaction between the long-tailed macaques and the general population in Singapore. As such, this study aims to determine the prevalence of simian malaria parasites in local macaques to assess the risk of zoonosis to the general human population. Screening for the presence of malaria parasites was conducted on blood samples from 660 peridomestic macaques collected between Jan 2008 and Mar 2017, and 379 wild macaques collected between Mar 2009 and Mar 2017, using a Pan-*Plasmodium*-genus specific PCR. Positive samples were then screened using a simian *Plasmodium* species-specific nested PCR assay to identify the species of parasites (*P*. *knowlesi*, *P*. *coatneyi*, *P*. *fieldi*, *P*. *cynomolgi*, and *P*. *inui)* present. All the peridomestic macaques sampled were tested negative for malaria, while 80.5% of the 379 wild macaques were infected. All five simian *Plasmodium* species were detected; *P*. *cynomolgi* being the most prevalent (71.5%), followed by *P*. *knowlesi* (47.5%), *P*. *inui* (42.0%), *P*. *fieldi* (32.5%), and *P*. *coatneyi* (28.5%). Co-infection with multiple species of *Plasmodium* parasites was also observed. The study revealed that Singapore’s wild long-tailed macaques are natural hosts of the five simian malaria parasite species, while no malaria was detected in all peridomestic macaques tested. Therefore, the risk of simian malaria transmission to the general human population is concluded to be low. However, this can be better demonstrated with the incrimination of the vectors of simian malaria parasites in Singapore.

## Author summary

Malaria is one of the most important vector-borne diseases in the world. In Southeast Asia, the incidence of human malaria infections caused by simian malaria parasites that originate from macaques has been increasing in the last decade, possibly due to closer proximity between the macaque and human population as a result of deforestation. In Singapore, urbanization and change in land use have resulted in closer proximity and interactions between macaques and the general human population. In order to assess the risk of potential simian malaria zoonosis, 1039 macaques’ blood samples were screened in this study for simian malaria parasites. Our results showed that simian malaria parasites were only detected in macaques living deep in the forest within the military protected zone, while macaques living close to human habitations are malaria-free. This study illustrated a low risk of zoonotic transmission of simian malaria parasites to the general population in Singapore.

## Introduction

Simian malaria parasites have been playing an increasing role in the human malaria burden [1], with *Plasmodium knowlesi* being an emerging zoonotic pathogen in many Southeast Asian countries [2–13]. The parasite is naturally found in long-tailed macaques (*Macaca fascicularis)* and pig-tailed macaques (*Macaca nemestrina)* [14]. As most malaria parasites are host-specific, naturally acquired *P*. *knowlesi* infections in humans were initially thought to be rare. However, a paradigm shift occurred in 2004 when a large cluster of human infections was detected in the Kapit division of Sarawak, East Malaysia [2]. These cases were initially misdiagnosed using microscopy as *P*. *knowlesi* is morphologically similar to *Plasmodium falciparum* and/or *Plasmodium malariae* species during the early ring and late trophozoite stages, respectively [2,15]. These cases were later confirmed to be *P*. *knowlesi* infection using molecular techniques. With an increased awareness of *P*. *knowlesi* infections in humans and the availability of determinative molecular assays, naturally acquired human knowlesi cases were subsequently reported in many parts of Southeast Asia: Myanmar [6,16], Laos [12], Vietnam [9,17], Cambodia [4], Thailand [8,18,19], Peninsular Malaysia [3], Singapore [13,20], Sabah in Malaysia Borneo [21], Indonesian Borneo [5,22], North Sumatra, Indonesia [23], and Philippines [7]. *Plasmodium knowlesi* has since been proposed as the fifth human malaria [2] and has been the most predominant cause of malaria in Malaysia [1,24,25].

Singapore reported its first naturally acquired human zoonotic malaria in 2007 [13]. The case was a military staff who contracted *P*. *knowlesi* infection after a period of training in a forested area in Singapore. This prompted a fever monitoring and surveillance programme for all military personnel who had spent periods of time in this area, and led to the detection of five additional cases of *P*. *knowlesi* infections; four cases in 2007 and one in 2008 [20,26]. All the infected persons were military personnel with no travel history but had spent time in the same forest prior to the onset of symptoms [20]. In the subsequent molecular epidemiological investigation of the knowlesi cases, Wong *et al* [20] also reported that three long-tailed macaques (*Macaca fascicularis*) caught in the forest where transmission was thought to occur, were infected with *P*. *knowlesi*. Phylogenetic analysis inferred from the *Plasmodium* circumsporozoite protein (*csp*) gene of the parasite revealed identical genotypes between the human cases and the infected macaques, suggesting that local human knowlesi cases had acquired the infection in the vicinity where these monkeys were found.

The finding of *P*. *knowlesi* in Singapore is of no surprise as this parasite was first discovered in a long-tailed macaque exported from Singapore to India in 1931 [27]. The recent report of *P*. *knowlesi* from long-tailed macaques, 80 years since its discovery, suggests the continuous and ongoing transmission of *P*. *knowlesi* in the local macaque population. The report of the locally acquired human knowlesi cases and the subsequent detection of *P*. *knowlesi* in wild macaques demonstrated the risk of zoonotic transmission of *P*. *knowlesi* in Singapore. Apart from *P*. *knowlesi*, long-tailed macaques in many Southeast Asian countries have shown to harbour *P*. *cynomolgi*, *P*. *inui*, *P*. *fieldi* and *P*. *coatneyi* [3,14,19,20,28–32], and the role of these macaques in contributing to the increased prevalence of zoonotic malaria in humans is evident. In a study conducted in the Hulu Selangor district of Selangor, Malaysia, a region where human *Plasmodium* infections are on a decline, the bulk of malaria infections were attributed to *P*. *knowlesi* [33]. Simian malaria prevalence study conducted on the macaques in this region revealed 50% of the sampled macaques were infected with *Plasmodium* parasites, of which 60% were *P*. *knowlesi* [28]. Deforestation has resulted in the migration of macaques to the forest fringes, alongside with the simiophagic mosquito vectors. With the majority of the human cases with knowlesi infections involved in the agricultural and forestry industries, likely, the increase in proximity between the macaques and the human population has led to increased zoonotic transmission of malaria [34,35].

Singapore is a small island state of area approximately 721.5 km^2^. Due to land scarcity, the majority of the forest cover had to make way for urbanization and industrialization. Currently, only the forests within the Bukit Timah Nature Reserve, Central Catchment Nature Reserve and the military training areas are the major forest reserves left. There are little buffer zones between the forest patches of the Nature reserve and human habitations, except for roads or highways separating the two. On the other hand, forest patches within the military training zone are generally away from the human settlement as it is a state-declared protected place with restricted access to the public.

*Macaca fascicularis*, the most predominant macaque species in Singapore, inhabits in the remaining patches of forests locally. In a population census report, it was noted that 70% of the macaque population resides in the Bukit Timah and Central Catchment Nature reserve [36,37]. Due to potential proximity and interactions between the macaques and the human population [38,39], this study aims to determine the prevalence of simian malaria parasites in Singapore’s long-tailed macaques, for evaluation of the risk of zoonotic transmission of simian malaria parasites to the general human population.

## Methods

### Ethics statement

Sample collection in this study was approved by the Singapore Armed Forces Joint Medical Committee and the DSO National Laboratory’s Institutional Animal Care and Use Committee.

### Macaque blood samples

Macaques in this surveillance study were categorized into two groups–“wild” and “peridomestic”. “Wild” macaques were caught in forested areas within the military protected zone in the Western Catchment area (see S1 Table), where local knowlesi cases were reported. As general public access to this patch of forest is restricted, macaques that reside in this forest were not exposed to human presence and food provision. The macaques from these areas were caught and sampled under an operational surveillance programme in response to the locally acquired human knowlesi case. Three hundred and seventy-nine wild macaques caught between Mar 2009 and March 2017 were screened for this study.

On the other hand, “peridomestic” macaques were found in close association with human habitations and were habituated to human presence and their food provision. Due to the closer macaque-human interactions and conflicts which arose, they were trapped and removed from various parts of Singapore (see S2 Table). Six hundred and sixty macaques caught between January 2008 and March 2017 were screened for this study.

**Fig 1:**
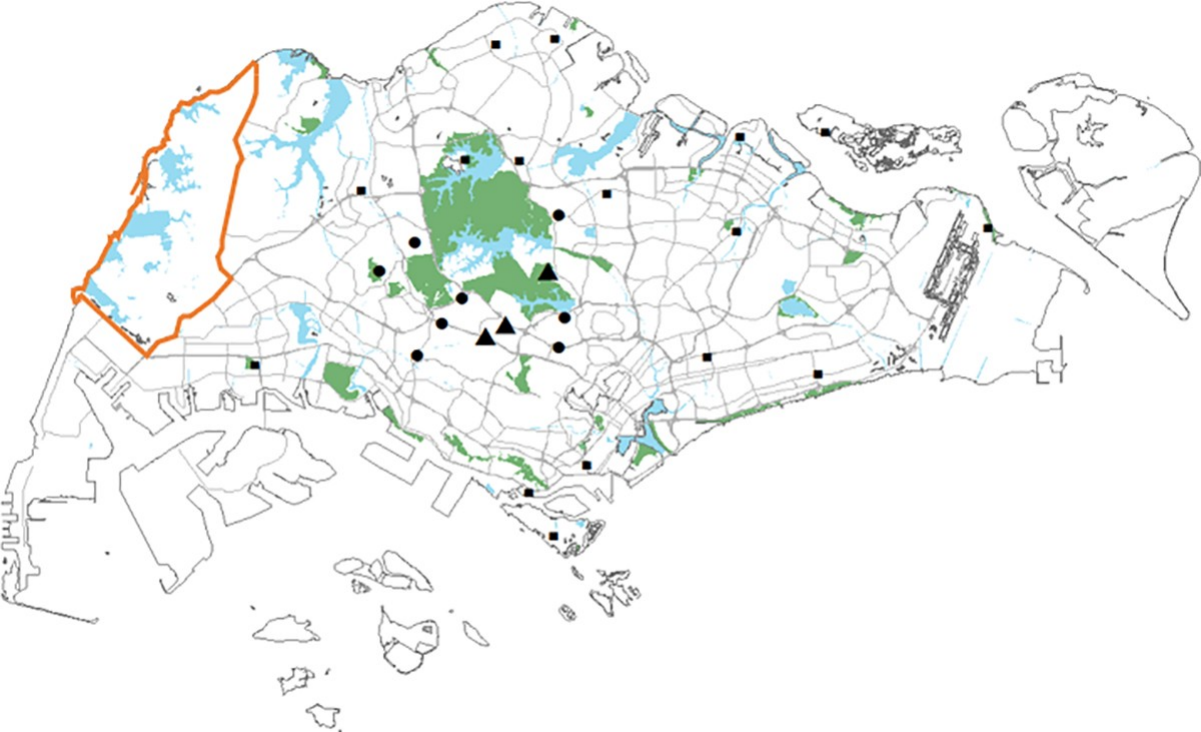
Distribution of macaques caught and screened for this study from Jan 2008 to Mar 2017. Western Catchment Area, the forest where all the wild macaques were trapped, is outlined in orange. Peridomestic macaque trapping locations were denoted by square, circle, and triangle for trapped macaque counts of less than 10, 10 to 49 and more than 50 respectively. (The map was downloaded from Onemap.sg).

Fig 1 illustrates the locations of macaques caught and screened in this study. Due to confidentiality, the exact locations of the macaques within the military protected area could not be revealed. Hence, the boundary of the Western Catchment Area was drawn to illustrate the region where wild macaques were trapped. On the other hand, the locations of the peridomestic macaques were aggregated based on the vicinity of the trapped locations.

All blood samples in this study were not collected systematically; the collection was based on convenience sampling from the two operational programmes. All macaques trapped were sent to Agri-Food and Veterinary Authority of Singapore (AVA) for age, sex, and blood sampling. The age of the macaques was estimated based on dentition [40]; those aged -three years and below were classified as juveniles while those estimated three years and above were classified as adults. All macaques were humanely euthanized by trained veterinarians in AVA prior to blood sampling, and blood samples collected in Ethylenediaminetetraacetic acid (EDTA) tubes were sent to Environmental Health Institute (EHI) for analysis. Blood was stored in -80°C until further use.

### DNA extraction

DNA was extracted from 200 μL of whole EDTA blood using DNeasy Blood and Tissue kit (QIAGEN, Hilden, Germany) according to the manufacturer’s instructions. Purified DNA was stored at −20°C until further use.

### Polymerase chain reaction (PCR) assay for detection of *Plasmodium* infection

Initial screening for the presence of malaria parasites was conducted using a Pan-*Plasmodium* PCR assay primers which target the 18S ribosomal RNA genes (PlasF: 5'—AGTGTGTATCAATCGAGTTTCT—3', PlasR: 5’—CTTGTCACTACCTCTCTTCTTTAGA -3’) [41]. PCR amplification was carried out in a 50 μL reaction volume, containing 5 μL of the genomic DNA as template, 1x reaction buffer (Promega, USA), 2.5 mM MgCl_2_ (Promega, USA), 200 mM of each dNTP (Promega, USA), 0.25 μM of each primer and 1.25U of GoTaq DNA polymerase (Promega, USA). The PCR was carried out using Veriti Thermal Cycler (Applied Biosystems, USA) with cycling conditions of 95°C for 4 min, 44 cycles of amplification at 95°C for 30 sec, 57°C for 30 sec, 72°C for 30 sec, followed by a final extension step of 2 min. Samples with *Plasmodium* parasites’ DNA will have an amplification product size of 188 bp. Summary of positive controls used in this study can be found in S3 Table.

### Nested PCR assays for simian malaria parasites speciation

For samples tested positive for malaria parasites, the species of *Plasmodium* parasites present were determined using published primers designed to target the 18S small sub-unit ribosomal RNA genes [2,41–43,44]. The nested PCR was conducted based on the assay described previously [41], with the exception that the nest 1 reaction was conducted in 50 μL volume.

All PCR reactions were carried out in Veriti Thermal Cycler (Applied Biosystems, USA) and PCR products were analyzed by 2% agarose gel electrophoresis.

### Statistical analysis

The linear trend of malaria parasites prevalence over the years was tested by regress function using STATA 14.2 (StataCorp, USA).

## Results

### Prevalence of malaria infection in macaques using Pan-*Plasmodium* PCR assay

*Plasmodium* screening conducted on 379 wild and 660 peridomestic long-tailed macaques revealed that 80.5% (305/379) of the wild macaques were infected with malaria parasites, while none of the 660 peridomestic macaques were infected (Table 1). The malaria prevalence rate in the adult and juvenile macaques was similar at 80.7% and 79.4% respectively.

**Table 1:** Summary of malaria infections in macaques sampled in this study.

	Wild macaques			Peridomestic macaques (N = 660)
	Adult (N = 305)	Juvenile (N = 73)	Undetermined (N = 1)	
Infected n (%)	246 (80.7)	58 (79.4)	1 (100)	0
Not infected n (%)	59 (19.3)	15 (20.5)	0 (0)	660 (100)
Total *Plasmodium* positive: 305 (all wild) Total *Plasmodium* negative: 734 (74 wild, 660 peridomestic) Total screened: 1039				

### Prevalence of simian malaria species using species-specific nested PCR assay

Using the published assay, all five simian malaria parasites were detected, with *P*. *cynomolgi* being the most prevalent (71.5%), followed by *P*. *knowlesi* (47.5%), *P*. *inui* (42.0%), *P*. *fieldi* (32.5%), and *P*. *coatneyi* (28.5%). Co-infection with multiple species of *Plasmodium* parasites was also observed; double infection was detected in 85 macaques, triple infection detected in 47 macaques, quadruple infection detected in 37, and quintuple infection detected in 23 macaques. Table 2 summarizes malaria infections in the infected macaques.

**Table 2:** Breakdown of malaria infections based on species-specific PCR assay in infected macaques.

Infection	*Plasmodium* species	Number of infections
Single	Pin	5
	Pk	30
	Pcy	60
	Pfi	1
	Pct	7
Double	Pk, Pin	3
	Pcy, Pin	25
	Pct, Pin	5
	Pfi, Pin	2
	Pk, Pcy	29
	Pct, Pcy	8
	Pfi, Pcy	7
	Pk, Pfi	2
	Pk, Pct	2
	Pct, Pfi	2
Triple	Pk, Pcy, Pin	6
	Pct, Pcy, Pin	7
	Pfi, Pcy, Pin	7
	Pk, Pin, Pfi	11
	Pk, Pcy, Pct	4
	Pfi, Pcy, Pct	5
	Pk, Pcy, Pfi	5
	Pk, Pct, Pin	1
	Pfi, Pin, Pct	1
Quadruple	Pcy, Pct, Pin, Pfi	8
	Pcy, Pk, Pin, Pfi	16
	Pcy, Pk, Pin, Pct	4
	Pcy, Pk, Pfi, Pct	5
	Pct, Pk, Pin, Pfi	4
Quintuple	Pct, Pk, Pin, Pfi, Pcy	23
**Total**		**295**

Pct, Pcy, Pfi, Pin, and Pk denotes *Plasmodium coatneyi*, *P*. *cynomolgi*, *P*. *fieldi*, *P*. *inui*, and *P*. *knowlesi*, respectively

**Fig 2:**
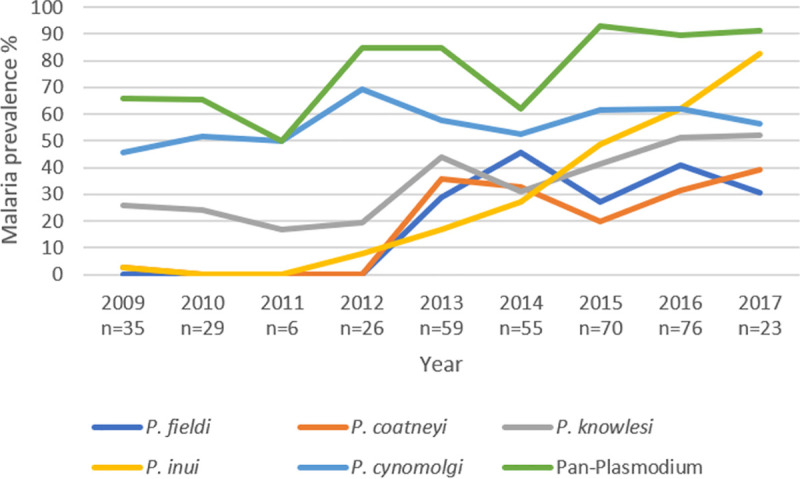
There is an increase in malaria prevalence among the sampled wild macaques from 2009 to 2017 (*p*-value for linear trend <0.0001). All species had a significant linear trend over the years (all only *p*<0.001) except for *P*. *cynomolgi*, which prevalence remained stable. The species of *Plasmodium* parasites were determined using previously reported primers designed to target the 18S small sub-unit ribosomal RNA genes [2,41–43].

The observed increasing trend of the proportion of infected macaques sampled across the years from 2009 to 2017 was found to be statistically significant. (Fig 2, *p*-value for linear trend *p*<0.001 for Pan-*Plasmodium*, *p*<0.001 for species-specific PCR). After stratifying by species of malaria, all species had a significant linear trend over the years (all only *p*<0.001) except for *P*. *cynomolgi*.

## Discussion

Our previous study revealed that wild long-tailed macaques were the reservoir hosts of locally-acquired human knowlesi infections [20]. As the previous study only included a small sample of macaques within the transmission area, this study aims to expand the number and geographical range of macaque samples to assess the risk of zoonotic transmission of simian malaria parasites to the general human population in Singapore. In this study, a convenience sampling design was used to collect macaques’ blood from the existing programmes.

Of the 1039 macaques screened in this study, all the malaria-infected macaques were caught in the forested grounds within the military protected area in the Western Catchment. Among these wild macaques, 80.5% (305/379) were tested positive using the Pan-*Plasmodium* PCR, while no malaria parasites were detected in all peridomestic macaques caught across different parts of Singapore. Similar observations were reported in Thailand and Malaysia [3,31], whereby all macaques caught in the urban areas were negative for malaria parasites while those caught from the forests had high infection rates. The absence of competent vectors in urban areas most likely explains the absence of malaria parasites in macaques caught in these areas [44].

Apart from *P*. *knowlesi*, *P*. *cynomolgi* and *P*. *inui* are also common malaria parasites found in the local macaque population, with 71.5% and 42.0% respectively. Naturally acquired *P*. *cynomolgi* infections have been reported in Peninsular Malaysia [10], Cambodia [11], and Malaysian Borneo [45,46], while *P*. *inui* is infectious to humans under laboratory studies [47]. In our study, the prevalence rate of *P*. *cynomolgi* is the highest, followed by *P*. *knowlesi*, *P*. *inui*, *P*. *fieldi* and then *P*. *coatneyi*. The prevalence obtained in a simian malaria study conducted in wild macaques from Selangor, Peninsular Malaysia was slightly different [28]. In the surveillance study, *P*. *inui* was the predominant (65.7%), followed by *P*. *knowlesi* (60%), *P*. *cynomolgi* (51.4%) *P*. *coatneyi* (45.7%) and *P*. *fieldi* (2.9%). Nevertheless, except for *P*. *fieldi* the other species are in the similar range with what we have observed in this study.

There was an increasing trend of the proportion of infected macaques sampled across the years from 2009 to 2017, based on both Pan-*Plasmodium* and simian malaria species-specific PCRs. This demonstrated an ongoing transmission of simian malaria parasites among the wild macaque population in the military protected forest within the Western Catchment area. The increasing trend was contributed by all species except for *P*. *cynomolgi*, which prevalence remained stable over the years. Apart from these five simian malaria species, there have been reports of *P*. *simiovale* and *P*. *inui*-like species detected in long-tailed macaques in Sarawak, Malaysian Borneo [48]. Hence, for future studies, it might be worthwhile to explore the use of advance molecular techniques like deep sequencing to detect novel simian malaria species.

Although macaques caught near human habitations were tested to be free from malaria infection, the possibility of wild macaque populations emigrating from the military protected forest within the Western Catchment area, alongside with the simiophagic mosquito vectors, cannot be ignored. This is particularly true in the land-scarce Singapore where residential areas may be built close to forest fringes and vegetation is cleared to meet increased demands for land use. As such, a thorough entomological surveillance targeting potential vectors of simian malaria is needed, particularly at the boundary of forests where housing estates are being built.

Despite the on-going transmission of malaria parasites among the wild macaques, the vectors involved in the transmission of zoonotic knowlesi in Singapore are yet to be identified. To date, only mosquitoes from the *Anopheles leucosphyrus* group have been incriminated in the transmission of *P*. *knowlesi* and other simian malaria parasites. These include *Anopheles hackeri* [49], *An*. *cracens* [3,50] in Peninsular Malaysia, *An*. *latens*, *An*. *donaldi* and *An*. *balabacensis* in Sarawak and Sabah of East Malaysia [51–54], and *An*. *dirus* in Vietnam [55,56]. Although Singapore lies within the distribution limit of the *Anopheles leucosphyrus* group [32], there have been no records on the presence of this species-group of mosquitoes locally [44,57].

The major elements in determining the suitability of control measures to be initiated highly depend on the identity and bionomics of the vectors found in the area [58]. Since the notification of the first human knowlesi case, intensive mosquito control activities such as the spraying of *Bacillus thuringiensis* var. *israelensis* and environmental control works were undertaken to eliminate mosquito breeding grounds within the forested grounds. Enhanced personal protection measures were enforced for all personnel training within these sites and these measures include the routine application of mosquito repellent on areas of exposed skin, the use of permethrin insecticide-treated uniforms and rolling down of long sleeves to prevent further transmission and infections [20]. These measures, which were implemented since 2007, had resulted in a significant reduction of total mosquito population from 64.1 mosquitoes per sampling site in August 2007 to 4.3 per site by June 2011 (Patrick Lam, pers comm, 14 November 2011). Since 2008, there had been no reported indigenous knowlesi cases in Singapore.

Despite the reduced mosquito population, the infection rate of wild macaques across the years remained high with an increased prevalence in recent years. There are a few plausible explanations for this. The trapping and removal of the macaques could have resulted in the reduction of their population in the Western Catchment area. The remaining macaque population would therefore been exposed to higher biting pressure, resulting in increased simian malaria transmission. In addition, the control measures taken to prevent human cases might not be effective against simiophagic vectors that are transmitting the infection among the macaques. As such, identifying the principal vector responsible for zoonotic knowlesi transmission is necessary for an appropriate vector control strategy to be developed. Another possibility for the observed increase in simian malaria prevalence over the years could be the degradation of DNA in samples collected in the earlier part of the study, resulting in the apparent lower prevalence rate detected in these samples.

The high malaria prevalence among the macaques suggests an active transmission among the macaque population in Western Catchment area. Therefore, to incriminate the vectors, mosquito surveillance should preferably include the use of monkey-baited traps [3,51] in addition to other entomological surveillance methods. The use of sentinel monkeys and determination of the home-range of the macaques may also aid in determining the high-risk transmission zone.

The main limitation of this study is the obtainment of samples through convenience sampling, which may result in sampling bias and inaccurate estimates. Therefore, the prevalence values for each species should be analysed with caution. Nonetheless, this study provided evidence that simian malaria infection found mainly in the macaque population from the Western Catchment forest, while no infection was detected among the peridomestic macaques sampled from other regions of Singapore which are accessible to the general public. As such, the risk of the general human population acquiring zoonotic malaria from the macaque is low.

## Conclusion

This study constitutes the first comprehensive report for surveillance of simian malaria parasites in long-tailed macaques in Singapore. In this study, we found that apart from *P*. *knowlesi*, wild long-tailed macaques also harbour *P*. *inui*, *P*. *cynomolgi*, *P*. *fieldi*, and *P*. *coatneyi*. Despite a high infection rate among the wild macaques, the risk of the general human population acquiring zoonotic malaria is low. However, the overall risk can be better demonstrated with information on the spatial distribution of macaques and the identification of vectors involved in the transmission among macaques and between macaques and humans.

## Supporting information

S1 Table List of wild macaques trapped within the military protected forest in the Western Catchment Area. (DOCX)

S2 Table List of peridomestic macaques trapped around Singapore (aggregated according to locations). (DOCX)

S3 Table Summary of positive controls used in the study. (DOCX)
