# Supplementary material for: Prevalence of simian malaria parasites in macaques of Singapore
Source: PLoS Negl Trop Dis. 2021 Jan 25;15(1):e0009110. doi: 10.1371/journal.pntd.0009110 (PMC7861519; doi:10.1371/journal.pntd.0009110)
Supplement: S1 Table — (DOCX) [file pntd.0009110.s001.docx]

S1 Table: List of wild macaques trapped within the military protected forest in the Western Catchment Area.

| **S/N.** | **Mth/Yr** | **Age** | **Gender** |
| --- | --- | --- | --- |
| 1 | Mar-09 | Juvenile | Male |
| 2 | Apr-09 | Adult | Male |
| 3 | Apr-09 | Adult | Male |
| 4 | Apr-09 | Juvenile | Male |
| 5 | Apr-09 | Juvenile | Male |
| 6 | Apr-09 | Juvenile | Female |
| 7 | Apr-09 | Juvenile | Male |
| 8 | Apr-09 | Juvenile | Male |
| 9 | Apr-09 | Juvenile | Male |
| 10 | Apr-09 | Adult | Female |
| 11 | Apr-09 | Juvenile | Male |
| 12 | May-09 | Adult | Female |
| 13 | Jun-09 | Juvenile | Female |
| 14 | Jul-09 | Juvenile | Female |
| 15 | Jul-09 | Adult | Male |
| 16 | Aug-09 | Adult | Female |
| 17 | Aug-09 | Juvenile | Female |
| 18 | Aug-09 | Adult | Male |
| 19 | Sep-09 | Adult | Female |
| 20 | Sep-09 | Juvenile | Female |
| 21 | Sep-09 | Adult | Male |
| 22 | Sep-09 | Adult | Male |
| 23 | Oct-09 | Adult | Male |
| 24 | Oct-09 | Adult | Female |
| 25 | Oct-09 | Adult | Male |
| 26 | Oct-09 | Adult | Male |
| 27 | Nov-09 | Adult | Female |
| 28 | Nov-09 | Adult | Male |
| 29 | Nov-09 | Adult | Male |
| 30 | Nov-09 | Juvenile | Male |
| 31 | Nov-09 | Juvenile | Female |
| 32 | Nov-09 | Adult | Male |
| 33 | Dec-09 | Adult | Male |
| 34 | Dec-09 | Adult | Male |
| 35 | Dec-09 | Adult | Female |
| 36 | Jan-10 | Adult | Male |
| 37 | Jan-10 | Adult | Female |
| 38 | Jan-10 | Adult | Male |
| 39 | Feb-10 | Juvenile | Male |
| 40 | Feb-10 | Adult | Male |
| 41 | Feb-10 | Adult | Male |
| 42 | Feb-10 | Juvenile | Male |
| 43 | Feb-10 | Adult | Female |
| 44 | Nov-10 | Adult | Female |
| 45 | Nov-10 | Adult | Male |
| 46 | Nov-10 | Adult | Male |
| 47 | Nov-10 | Juvenile | Male |
| 48 | Nov-10 | Adult | Female |
| 49 | Nov-10 | Juvenile | Female |
| 50 | Nov-10 | Adult | Female |
| 51 | Nov-10 | Adult | Female |
| 52 | Dec-10 | Adult | Male |
| 53 | Dec-10 | Adult | Male |
| 54 | Dec-10 | Adult | Female |
| 55 | Dec-10 | Adult | Female |
| 56 | Dec-10 | Adult | Male |
| 57 | Dec-10 | Adult | Male |
| 58 | Dec-10 | Adult | Female |
| 59 | Dec-10 | Adult | Female |
| 60 | Dec-10 | Adult | Female |
| 61 | Dec-10 | Adult | Male |
| 62 | Dec-10 | Juvenile | Male |
| 63 | Dec-10 | Adult | Female |
| 64 | Dec-10 | Adult | Female |
| 65 | Jan-11 | Adult | Female |
| 66 | Jan-11 | Adult | Female |
| 67 | Jan-11 | Juvenile | Female |
| 68 | Jan-11 | Juvenile | Male |
| 69 | Jan-11 | Adult | Male |
| 70 | Jan-11 | Juvenile | Female |
| 71 | Feb-12 | Juvenile | Male |
| 72 | Jun-12 | Adult | Female |
| 73 | Jun-12 | Adult | Male |
| 74 | Jun-12 | Adult | Male |
| 75 | Jun-12 | Adult | Male |
| 76 | Mar-12 | Adult | Female |
| 77 | Mar-12 | Juvenile | Male |
| 78 | Mar-12 | Adult | Male |
| 79 | Mar-12 | Adult | Female |
| 80 | Mar-12 | Adult | Male |
| 81 | Mar-12 | Adult | Male |
| 82 | Mar-12 | Juvenile | Male |
| 83 | Apr-12 | Adult | Male |
| 84 | May-12 | Adult | Male |
| 85 | Nov-12 | Juvenile | Male |
| 86 | Jun-12 | Adult | Male |
| 87 | Jun-12 | Juvenile | Female |
| 88 | Jun-12 | Juvenile | Female |
| 89 | Jun-12 | Adult | Female |
| 90 | Jun-12 | Adult | Female |
| 91 | Jun-12 | Juvenile | Male |
| 92 | Jun-12 | Juvenile | Male |
| 93 | Oct-12 | Adult | Male |
| 94 | Jul-12 | Adult | Female |
| 95 | Oct-12 | Adult | Male |
| 96 | Oct-12 | Adult | Male |
| 97 | Apr-13 | Juvenile | Male |
| 98 | Apr-13 | Adult | Male |
| 99 | Apr-13 | Adult | Female |
| 100 | Mar-13 | Adult | Female |
| 101 | Mar-13 | Adult | Male |
| 102 | Mar-13 | Adult | Male |
| 103 | Mar-13 | Adult | Female |
| 104 | Mar-13 | Adult | Male |
| 105 | Mar-13 | Adult | Female |
| 106 | Jul-13 | Adult | Male |
| 107 | Oct-13 | Adult | Male |
| 108 | Oct-13 | Adult | Male |
| 109 | Oct-13 | Adult | Male |
| 110 | Oct-13 | Adult | Male |
| 111 | May-13 | Adult | Male |
| 112 | May-13 | Adult | Male |
| 113 | May-13 | Juvenile | Female |
| 114 | May-13 | Juvenile | Female |
| 115 | May-13 | Adult | Female |
| 116 | May-13 | Adult | Male |
| 117 | May-13 | Adult | Female |
| 118 | May-13 | Juvenile | Female |
| 119 | May-13 | Adult | Female |
| 120 | May-13 | Juvenile | Male |
| 121 | Apr-13 | Adult | Female |
| 122 | Apr-13 | Adult | Female |
| 123 | Apr-13 | Adult | Male |
| 124 | Apr-13 | Adult | Female |
| 125 | Jul-13 | Adult | Male |
| 126 | Jul-13 | Juvenile | Male |
| 127 | Jun-13 | Adult | Female |
| 128 | Jun-13 | Adult | Female |
| 129 | May-13 | Adult | Male |
| 130 | May-13 | Adult | Female |
| 131 | May-13 | Adult | Female |
| 132 | Sep-13 | Adult | Male |
| 133 | Dec-13 | Juvenile | Male |
| 134 | Jul-13 | Adult | Female |
| 135 | Jul-13 | Adult | Male |
| 136 | Jul-13 | Adult | Female |
| 137 | Jul-13 | Adult | Female |
| 138 | Jul-13 | Adult | Female |
| 139 | Aug-13 | Adult | Male |
| 140 | Aug-13 | Adult | Female |
| 141 | Aug-13 | Adult | Female |
| 142 | Aug-13 | Adult | Female |
| 143 | Jun-13 | Adult | Male |
| 144 | Oct-13 | Adult | Female |
| 145 | Oct-13 | Adult | Male |
| 146 | Sep-13 | Adult | Male |
| 147 | Oct-13 | Juvenile | Male |
| 148 | Oct-13 | Adult | Male |
| 149 | Nov-13 | Adult | Male |
| 150 | Nov-13 | Adult | Male |
| 151 | Oct-13 | Adult | Male |
| 152 | Oct-13 | Adult | Male |
| 153 | Dec-13 | Adult | Female |
| 154 | Dec-13 | Adult | Female |
| 155 | Dec-13 | Adult | Female |
| 156 | Jan-14 | Adult | Male |
| 157 | Jan-14 | Adult | Female |
| 158 | Jul-14 | Adult | Female |
| 159 | Feb-14 | Adult | Female |
| 160 | Feb-14 | Adult | Male |
| 161 | Feb-14 | Adult | Female |
| 162 | Apr-14 | Adult | Female |
| 163 | Apr-14 | Adult | Male |
| 164 | Jul-14 | Adult | Female |
| 165 | Nov-14 | Adult | Male |
| 166 | Mar-14 | Adult | Male |
| 167 | Mar-14 | Adult | Female |
| 168 | Mar-14 | Adult | Male |
| 169 | Oct-14 | Juvenile | Female |
| 170 | Apr-14 | Adult | Female |
| 171 | Dec-14 | Adult | Male |
| 172 | May-14 | Adult | Female |
| 173 | Sep-14 | Juvenile | Male |
| 174 | Mar-14 | Adult | Male |
| 175 | Mar-14 | Juvenile | Male |
| 176 | Oct-14 | Adult | Male |
| 177 | Oct-14 | Adult | Male |
| 178 | Oct-14 | Adult | Male |
| 179 | Oct-14 | Adult | Male |
| 180 | Oct-14 | Adult | Male |
| 181 | Oct-14 | Adult | Male |
| 182 | Oct-14 | Adult | Female |
| 183 | May-14 | Adult | Male |
| 184 | Nov-14 | Adult | Female |
| 185 | Nov-14 | Adult | Female |
| 186 | Nov-14 | Adult | Female |
| 187 | Nov-14 | Adult | Female |
| 188 | Nov-14 | Juvenile | Male |
| 189 | May-14 | Juvenile | Male |
| 190 | Oct-14 | Juvenile | Male |
| 191 | Oct-14 | Adult | Female |
| 192 | Dec-14 | Adult | Male |
| 193 | Dec-14 | Adult | Male |
| 194 | Dec-14 | Adult | Female |
| 195 | Dec-14 | Adult | Female |
| 196 | Dec-14 | Adult | Female |
| 197 | Dec-14 | Adult | Female |
| 198 | Dec-14 | Adult | Female |
| 199 | Dec-14 | Adult | Male |
| 200 | Dec-14 | Juvenile | Male |
| 201 | Dec-14 | Juvenile | Male |
| 202 | Dec-14 | Adult | Female |
| 203 | Dec-14 | Adult | Male |
| 204 | Feb-15 | Adult | Male |
| 205 | Feb-15 | Adult | Female |
| 206 | Jun-15 | Adult | Female |
| 207 | Jan-14 | Adult | Female |
| 208 | Jan-14 | Adult | Female |
| 209 | Jan-14 | Adult | Male |
| 210 | Jan-14 | Adult | Female |
| 211 | Jan-15 | Adult | Female |
| 212 | Jan-15 | Adult | Male |
| 213 | Jan-15 | Adult | Female |
| 214 | Feb-15 | Adult | Male |
| 215 | Feb-15 | Adult | Female |
| 216 | Apr-15 | Adult | Male |
| 217 | Apr-15 | Adult | Male |
| 218 | Apr-15 | Juvenile | Female |
| 219 | Apr-15 | Adult | Female |
| 220 | Apr-15 | Juvenile | Male |
| 221 | Apr-15 | Juvenile | Male |
| 222 | Apr-15 | Juvenile | Female |
| 223 | Apr-15 | Adult | Male |
| 224 | Apr-15 | Adult | Male |
| 225 | May-15 | Adult | Male |
| 226 | May-15 | Juvenile | Female |
| 227 | Dec-15 | Adult | Female |
| 228 | Dec-15 | Adult | Male |
| 229 | Jun-15 | Juvenile | Male |
| 230 | Jun-15 | Adult | Male |
| 231 | Jun-15 | Adult | Female |
| 232 | Jun-15 | Adult | Male |
| 233 | Jun-15 | Adult | Male |
| 234 | Jun-15 | Adult | Male |
| 235 | Jun-15 | Adult | Male |
| 236 | Jul-15 | Juvenile | Male |
| 237 | Jul-15 | Juvenile | Male |
| 238 | Jul-15 | Juvenile | Female |
| 239 | Jul-15 | Adult | Male |
| 240 | Jun-15 | Adult | Female |
| 241 | Jun-15 | Juvenile | Female |
| 242 | Jun-15 | Juvenile | Female |
| 243 | Jun-15 | Adult | Female |
| 244 | Jun-15 | Adult | Female |
| 245 | Aug-15 | Adult | Male |
| 246 | Aug-15 | Adult | Male |
| 247 | Apr-15 | Adult | Female |
| 248 | Apr-15 | Adult | Male |
| 249 | Apr-15 | Adult | Male |
| 250 | Apr-15 | N | N |
| 251 | Apr-15 | Adult | Male |
| 252 | Aug-15 | Adult | Male |
| 253 | Aug-15 | Adult | Male |
| 254 | Sep-15 | Juvenile | Male |
| 255 | Sep-15 | Adult | Female |
| 256 | Sep-15 | Adult | Male |
| 257 | Sep-15 | Adult | Male |
| 258 | Feb-15 | Adult | Female |
| 259 | Feb-15 | Adult | Female |
| 260 | Feb-15 | Adult | Female |
| 261 | Feb-15 | Adult | Female |
| 262 | Sep-15 | Adult | Female |
| 263 | Oct-15 | Adult | Female |
| 264 | Oct-15 | Juvenile | Male |
| 265 | Oct-15 | Adult | Female |
| 266 | Feb-15 | Adult | Male |
| 267 | Feb-15 | Adult | Male |
| 268 | Nov-15 | Adult | Female |
| 269 | Nov-15 | Adult | Female |
| 270 | Nov-15 | Adult | Female |
| 271 | Nov-15 | Adult | Female |
| 272 | Nov-15 | Juvenile | Male |
| 273 | Nov-15 | Juvenile | Male |
| 274 | Nov-15 | Juvenile | Male |
| 275 | Mar-15 | Juvenile | Male |
| 276 | Mar-15 | Juvenile | Male |
| 277 | Mar-15 | Adult | Female |
| 278 | Nov-15 | Adult | Female |
| 279 | Dec-15 | Juvenile | Female |
| 280 | Dec-15 | Adult | Female |
| 281 | Jun-16 | Adult | Male |
| 282 | Jan-16 | Adult | Male |
| 283 | Jan-16 | Adult | Male |
| 284 | Jan-16 | Adult | Female |
| 285 | Jan-16 | Adult | Female |
| 286 | Feb-16 | Adult | Male |
| 287 | Feb-16 | Adult | Female |
| 288 | Feb-16 | Adult | Male |
| 289 | Feb-16 | Adult | Female |
| 290 | Apr-16 | Adult | Female |
| 291 | Apr-16 | Juvenile | Female |
| 292 | Nov-16 | Adult | Male |
| 293 | Mar-16 | Juvenile | Male |
| 294 | Mar-16 | Adult | Male |
| 295 | Jan-16 | Juvenile | Female |
| 296 | Jan-16 | Adult | Female |
| 297 | Jan-16 | Adult | Male |
| 298 | Jan-16 | Adult | Female |
| 299 | Apr-16 | Adult | Male |
| 300 | Aug-16 | Adult | Female |
| 301 | Apr-16 | Juvenile | Female |
| 302 | Apr-16 | Adult | Female |
| 303 | Apr-16 | Adult | Female |
| 304 | Apr-16 | Adult | Female |
| 305 | Apr-16 | Juvenile | Male |
| 306 | May-16 | Adult | Male |
| 307 | May-16 | Adult | Male |
| 308 | May-16 | Adult | Male |
| 309 | May-16 | Adult | Female |
| 310 | May-16 | Adult | Male |
| 311 | May-16 | Adult | Male |
| 312 | Jan-16 | Adult | Female |
| 313 | Jan-16 | Adult | Male |
| 314 | Jun-16 | Adult | Female |
| 315 | Jun-16 | Adult | Female |
| 316 | Jan-16 | Adult | Male |
| 317 | Jan-16 | Adult | Male |
| 318 | Jul-16 | Adult | Male |
| 319 | Jul-16 | Adult | Male |
| 320 | Jul-16 | Adult | Male |
| 321 | Jul-16 | Adult | Male |
| 322 | Jul-16 | Adult | Male |
| 323 | Jul-16 | Adult | Male |
| 324 | Jul-16 | Adult | Male |
| 325 | Jul-16 | Adult | Male |
| 326 | Feb-16 | Adult | Male |
| 327 | Feb-16 | Juvenile | Male |
| 328 | Apr-16 | Adult | Male |
| 329 | Apr-16 | Adult | Male |
| 330 | Oct-16 | Adult | Female |
| 331 | Oct-16 | Adult | Male |
| 332 | Feb-16 | Adult | Female |
| 333 | Feb-16 | Adult | Male |
| 334 | Jun-16 | Adult | Male |
| 335 | Sep-16 | Adult | Male |
| 336 | Sep-16 | Adult | Female |
| 337 | Sep-16 | Adult | Female |
| 338 | Sep-16 | Adult | Female |
| 339 | Sep-16 | Adult | Male |
| 340 | Sep-16 | Adult | Female |
| 341 | Sep-16 | Adult | Male |
| 342 | Sep-16 | Adult | Female |
| 343 | Oct-16 | Adult | Male |
| 344 | Oct-16 | Adult | Male |
| 345 | Oct-16 | Adult | Male |
| 346 | Oct-16 | Adult | Male |
| 347 | Oct-16 | Adult | Male |
| 348 | Jan-16 | Adult | Male |
| 349 | Nov-16 | Juvenile | Female |
| 350 | Dec-16 | Adult | Female |
| 351 | Dec-16 | Adult | Male |
| 352 | Dec-16 | Adult | Male |
| 353 | Dec-16 | Adult | Female |
| 354 | Dec-16 | Juvenile | Male |
| 355 | Dec-16 | Adult | Male |
| 356 | Mar-16 | Adult | Female |
| 357 | Apr-17 | Adult | Male |
| 358 | Apr-17 | Adult | Male |
| 359 | Jun-17 | Adult | Female |
| 360 | Jun-17 | Adult | Female |
| 361 | Jan-17 | Adult | Male |
| 362 | Jan-17 | Adult | Male |
| 363 | Jan-17 | Adult | Male |
| 364 | Jan-17 | Adult | Male |
| 365 | Jan-17 | Adult | Male |
| 366 | Jan-17 | Adult | Male |
| 367 | Jan-17 | Adult | Female |
| 368 | Jan-17 | Adult | Male |
| 369 | Feb-17 | Adult | Male |
| 370 | Feb-17 | Adult | Male |
| 371 | Feb-17 | Adult | Male |
| 372 | Feb-17 | Adult | Female |
| 373 | Mar-17 | Adult | Male |
| 374 | Mar-17 | Adult | Female |
| 375 | Mar-17 | Adult | Female |
| 376 | Mar-17 | Adult | Male |
| 377 | Mar-17 | Adult | Female |
| 378 | Mar-17 | Adult | Male |
| 379 | Mar-17 | Adult | Male |
