# Supplementary material for: Prevalence of simian malaria parasites in macaques of Singapore
Source: PLoS Negl Trop Dis. 2021 Jan 25;15(1):e0009110. doi: 10.1371/journal.pntd.0009110 (PMC7861519; doi:10.1371/journal.pntd.0009110)
Supplement: S2 Table — (DOCX) [file pntd.0009110.s002.docx]

S2 Table: List of peridomestic macaques trapped around Singapore (aggregated according to locations)

| **S/N.** | **Mth/Yr** | **Age** | **Gender** | **Count** |
| --- | --- | --- | --- | --- |
| 1 | Sep-16 | Juvenile | Male | 16 |
| 2 | Jun-16 | Adult | Male |  |
| 3 | Jun-16 | Adult | Male |  |
| 4 | Jun-16 | Adult | Female |  |
| 5 | Jun-16 | Adult | Male |  |
| 6 | Aug-16 | Adult | Female |  |
| 7 | Sep-16 | Adult | Male |  |
| 8 | Sep-16 | Adult | Female |  |
| 9 | Sep-16 | Adult | Male |  |
| 10 | Sep-16 | Adult | Female |  |
| 11 | Sep-16 | Adult | Female |  |
| 12 | Sep-16 | Adult | Male |  |
| 13 | Sep-16 | Adult | Female |  |
| 14 | Sep-16 | Adult | Female |  |
| 15 | Sep-16 | Adult | Female |  |
| 16 | Nov-16 | Adult | Male |  |
| 17 | Mar-10 | Adult | Male | 94 |
| 18 | Mar-10 | Adult | Male |  |
| 19 | Mar-10 | Juvenile | Male |  |
| 20 | Mar-10 | Adult | Male |  |
| 21 | Mar-10 | Adult | Male |  |
| 22 | Aug-10 | Adult | Male |  |
| 23 | Jan-16 | Adult | Female |  |
| 24 | Feb-16 | Adult | Female |  |
| 25 | May-16 | Adult | Male |  |
| 26 | May-16 | Adult | Male |  |
| 27 | Jun-16 | Adult | Male |  |
| 28 | Jun-16 | Adult | Male |  |
| 29 | Jun-16 | Adult | Male |  |
| 30 | Jun-16 | Adult | Female |  |
| 31 | Oct-16 | Juvenile | Male |  |
| 32 | Oct-16 | Adult | Female |  |
| 33 | Nov-16 | Adult | Female |  |
| 34 | Nov-16 | Adult | Male |  |
| 35 | Dec-16 | Adult | Female |  |
| 36 | Dec-16 | Juvenile | Male |  |
| 37 | Dec-16 | Adult | Female |  |
| 38 | Dec-16 | Juvenile | Female |  |
| 39 | Apr-11 | Adult | Female |  |
| 40 | Jan-15 | Adult | Female |  |
| 41 | Jan-15 | Adult | Male |  |
| 42 | Jan-15 | Adult | Male |  |
| 43 | Jan-15 | Adult | Female |  |
| 44 | Jan-15 | Adult | Female |  |
| 45 | May-16 | Adult | Male |  |
| 46 | May-16 | Adult | Female |  |
| 47 | May-16 | Juvenile | Female |  |
| 48 | May-16 | Adult | Female |  |
| 49 | Jun-16 | Juvenile | Female |  |
| 50 | Jun-16 | Juvenile | Female |  |
| 51 | Jun-16 | Adult | Female |  |
| 52 | Jun-16 | Juvenile | Female |  |
| 53 | Jun-16 | Juvenile | Male |  |
| 54 | Sep-16 | Adult | Male |  |
| 55 | Sep-16 | Adult | Female |  |
| 56 | Mar-15 | Adult | Male |  |
| 57 | Jul-15 | Juvenile | Female |  |
| 58 | Jul-15 | Juvenile | Female |  |
| 59 | Sep-15 | Adult | Female |  |
| 60 | Sep-15 | Adult | Male |  |
| 61 | Sep-15 | Adult | Male |  |
| 62 | Sep-15 | Juvenile | Male |  |
| 63 | Oct-15 | Adult | Female |  |
| 64 | Oct-15 | Adult | Female |  |
| 65 | Oct-15 | Adult | Male |  |
| 66 | Feb-16 | Juvenile | Male |  |
| 67 | Mar-16 | Adult | Male |  |
| 68 | May-16 | Adult | Male |  |
| 69 | May-16 | Juvenile | Female |  |
| 70 | Jan-17 | Juvenile | Male |  |
| 71 | Jan-17 | Adult | Female |  |
| 72 | Jan-17 | Adult | Male |  |
| 73 | Jan-17 | Adult | Male |  |
| 74 | Feb-17 | Juvenile | Female |  |
| 75 | Feb-17 | Adult | Female |  |
| 76 | Feb-16 | Adult | Female |  |
| 77 | Feb-16 | Adult | Female |  |
| 78 | Feb-16 | Adult | Male |  |
| 79 | Mar-16 | Adult | Female |  |
| 80 | May-16 | Juvenile | Male |  |
| 81 | Jul-16 | Adult | Male |  |
| 82 | Jul-16 | Adult | Female |  |
| 83 | Aug-16 | Adult | Male |  |
| 84 | Dec-16 | Adult | Female |  |
| 85 | Dec-16 | Adult | Female |  |
| 86 | Dec-16 | Adult | Male |  |
| 87 | Jan-17 | Adult | Female |  |
| 88 | Jan-17 | Adult | Female |  |
| 89 | Jan-17 | Adult | Male |  |
| 90 | Feb-17 | Adult | Male |  |
| 91 | Aug-15 | Juvenile | Female |  |
| 92 | Feb-10 | Adult | Male |  |
| 93 | Apr-11 | Adult | Male |  |
| 94 | Apr-11 | Adult | Male |  |
| 95 | Apr-11 | Adult | Male |  |
| 96 | Apr-11 | Juvenile | Male |  |
| 97 | Apr-11 | Adult | Male |  |
| 98 | Feb-16 | Juvenile | Female |  |
| 99 | May-16 | Adult | Male |  |
| 100 | Jan-08 | Adult | Male |  |
| 101 | Jan-08 | Adult | Male |  |
| 102 | Jan-08 | Adult | Male |  |
| 103 | Jan-08 | Adult | Male |  |
| 104 | Jan-08 | Adult | Male |  |
| 105 | Jan-08 | Adult | Male |  |
| 106 | Jan-08 | Adult | Male |  |
| 107 | Jan-08 | Adult | Male |  |
| 108 | Jan-08 | Adult | Male |  |
| 109 | Jan-08 | Adult | Male |  |
| 110 | Mar-10 | Juvenile | Male |  |
| 111 | Feb-15 | Adult | Female | 46 |
| 112 | Feb-15 | Adult | Male |  |
| 113 | Mar-15 | Adult | Male |  |
| 114 | Mar-15 | Adult | Male |  |
| 115 | Apr-15 | Adult | Female |  |
| 116 | Jul-15 | Juvenile | Male |  |
| 117 | Jul-15 | Adult | Female |  |
| 118 | Sep-15 | Juvenile | Female |  |
| 119 | Sep-15 | Juvenile | Male |  |
| 120 | Sep-15 | Adult | Male |  |
| 121 | Oct-15 | Adult | Male |  |
| 122 | Oct-15 | Juvenile | Male |  |
| 123 | Nov-15 | Adult | Female |  |
| 124 | Nov-15 | Adult | Male |  |
| 125 | Nov-15 | Juvenile | Male |  |
| 126 | Nov-15 | Adult | Male |  |
| 127 | Nov-15 | Adult | Female |  |
| 128 | Nov-15 | Juvenile | Female |  |
| 129 | Aug-16 | Adult | Male |  |
| 130 | Aug-15 | Adult | Male |  |
| 131 | Aug-15 | Adult | Male |  |
| 132 | Aug-15 | Adult | Male |  |
| 133 | Oct-15 | Adult | Male |  |
| 134 | Oct-15 | Adult | Male |  |
| 135 | Feb-11 | Juvenile | Male |  |
| 136 | Jun-16 | Juvenile | Female |  |
| 137 | Sep-16 | Juvenile | Female |  |
| 138 | Sep-16 | Adult | Female |  |
| 139 | Jun-15 | Adult | Male |  |
| 140 | Jun-15 | Adult | Male |  |
| 141 | Aug-15 | Juvenile | Male |  |
| 142 | Sep-15 | Adult | Female |  |
| 143 | Oct-15 | Adult | Female |  |
| 144 | Nov-15 | Adult | Male |  |
| 145 | Aug-15 | Juvenile | Female |  |
| 146 | Oct-15 | Adult | Male |  |
| 147 | Oct-15 | Adult | Female |  |
| 148 | Oct-15 | Adult | Female |  |
| 149 | May-16 | Adult | Male |  |
| 150 | Jan-15 | Adult | Male |  |
| 151 | Jan-15 | Adult | Male |  |
| 152 | Jun-16 | Adult | Female |  |
| 153 | Jun-16 | Adult | Female |  |
| 154 | Aug-16 | Adult | Female |  |
| 155 | Aug-16 | Adult | Female |  |
| 156 | Apr-10 | Adult | Male |  |
| 157 | Apr-15 | Juvenile | Male | 246 |
| 158 | Apr-15 | Juvenile | Male |  |
| 159 | Apr-15 | Adult | Male |  |
| 160 | Apr-15 | Juvenile | Male |  |
| 161 | Apr-15 | Juvenile | Male |  |
| 162 | Jun-15 | Adult | Female |  |
| 163 | Feb-17 | Juvenile | Male |  |
| 164 | Feb-17 | Adult | Female |  |
| 165 | Feb-17 | Adult | Male |  |
| 166 | Mar-17 | Adult | Female |  |
| 167 | Mar-17 | Adult | Male |  |
| 168 | Mar-17 | Juvenile | Male |  |
| 169 | Mar-17 | Adult | Female |  |
| 170 | Feb-16 | Adult | Female |  |
| 171 | Dec-16 | Adult | Female |  |
| 172 | Sep-15 | Juvenile | Female |  |
| 173 | Sep-15 | Adult | Female |  |
| 174 | Feb-16 | Juvenile | Male |  |
| 175 | Feb-16 | Juvenile | Male |  |
| 176 | Jun-15 | Adult | Female |  |
| 177 | Jul-15 | Adult | Male |  |
| 178 | Jul-15 | Adult | Female |  |
| 179 | Oct-15 | Adult | Male |  |
| 180 | Oct-15 | Adult | Male |  |
| 181 | Oct-15 | Adult | Female |  |
| 182 | Nov-15 | Adult | Male |  |
| 183 | Dec-15 | Adult | Male |  |
| 184 | Feb-16 | Adult | Female |  |
| 185 | May-16 | Juvenile | Male |  |
| 186 | May-16 | Adult | Female |  |
| 187 | May-16 | Adult | Female |  |
| 188 | Jul-16 | Adult | Female |  |
| 189 | Aug-16 | Adult | Male |  |
| 190 | Sep-16 | Juvenile | Female |  |
| 191 | Sep-16 | Adult | Male |  |
| 192 | Dec-16 | Adult | Male |  |
| 193 | Dec-16 | Juvenile | Female |  |
| 194 | Dec-16 | Adult | Female |  |
| 195 | Dec-16 | Adult | Male |  |
| 196 | Jan-17 | Adult | Female |  |
| 197 | Jan-17 | Adult | Female |  |
| 198 | Jan-17 | Adult | Male |  |
| 199 | Oct-10 | Adult | Male |  |
| 200 | Feb-15 | Adult | Male |  |
| 201 | Feb-15 | Adult | Male |  |
| 202 | May-15 | Juvenile | Male |  |
| 203 | Jun-15 | Adult | Female |  |
| 204 | Jul-15 | Juvenile | Female |  |
| 205 | Jul-15 | Juvenile | Male |  |
| 206 | Jul-15 | Juvenile | Female |  |
| 207 | Jul-15 | Juvenile | Male |  |
| 208 | Jul-15 | Juvenile | Female |  |
| 209 | Aug-15 | Adult | Female |  |
| 210 | Aug-15 | Juvenile | Male |  |
| 211 | Aug-15 | Adult | Female |  |
| 212 | Aug-15 | Juvenile | Female |  |
| 213 | Aug-15 | Adult | Female |  |
| 214 | Aug-15 | Adult | Female |  |
| 215 | Aug-15 | Adult | Female |  |
| 216 | Sep-15 | Adult | Male |  |
| 217 | Sep-15 | Juvenile | Male |  |
| 218 | Oct-15 | Adult | Male |  |
| 219 | Oct-15 | Adult | Male |  |
| 220 | Oct-15 | Adult | Female |  |
| 221 | Oct-15 | Adult | Female |  |
| 222 | Oct-15 | Adult | Male |  |
| 223 | Oct-15 | Juvenile | Female |  |
| 224 | Oct-15 | Adult | Female |  |
| 225 | Nov-15 | Adult | Male |  |
| 226 | Nov-15 | Adult | Male |  |
| 227 | Nov-15 | Adult | Male |  |
| 228 | Nov-15 | Adult | Male |  |
| 229 | Jan-16 | Juvenile | Male |  |
| 230 | Jan-16 | Adult | Female |  |
| 231 | Jan-16 | Adult | Male |  |
| 232 | Feb-16 | Adult | Male |  |
| 233 | Feb-16 | Adult | Male |  |
| 234 | Mar-16 | Juvenile | Male |  |
| 235 | May-16 | Adult | Male |  |
| 236 | May-16 | Adult |  |  |
| 237 | May-16 | Adult | Female |  |
| 238 | Jun-16 | Adult | Male |  |
| 239 | Aug-16 | Adult | Male |  |
| 240 | Sep-16 | Adult | Female |  |
| 241 | Oct-16 | Adult | Male |  |
| 242 | Nov-16 | Adult | Male |  |
| 243 | Dec-16 | Adult | Female |  |
| 244 | Dec-16 | Adult | Male |  |
| 245 | Dec-16 | Adult | Male |  |
| 246 | Dec-16 | Adult | Female |  |
| 247 | Jan-17 | Adult | Female |  |
| 248 | Jan-17 | Adult | Female |  |
| 249 | Feb-17 | Adult | Male |  |
| 250 | Feb-17 | Adult | Female |  |
| 251 | Oct-15 | Juvenile | Male |  |
| 252 | Feb-11 | Adult | Female |  |
| 253 | Jul-15 | Adult | Female |  |
| 254 | Jul-15 | Adult | Female |  |
| 255 | Oct-15 | Juvenile | Female |  |
| 256 | Oct-15 | Juvenile | Female |  |
| 257 | Oct-15 | Adult | Male |  |
| 258 | Oct-15 | Adult | Male |  |
| 259 | Oct-15 | Adult | Female |  |
| 260 | Oct-15 | Adult | Male |  |
| 261 | Nov-15 | Adult | Male |  |
| 262 | Feb-16 | Juvenile | Male |  |
| 263 | Feb-16 | Adult | Male |  |
| 264 | Feb-16 | Adult | Male |  |
| 265 | Feb-16 | Adult | Male |  |
| 266 | Feb-16 | Juvenile | Male |  |
| 267 | May-16 | Adult | Male |  |
| 268 | May-16 | Adult | Male |  |
| 269 | May-16 | Adult | Male |  |
| 270 | May-16 | Adult | Female |  |
| 271 | Jun-16 | Adult | Male |  |
| 272 | Jun-16 | Adult | Male |  |
| 273 | Jun-16 | Adult | Male |  |
| 274 | Jun-16 | Adult | Female |  |
| 275 | Jul-16 | Juvenile | Male |  |
| 276 | Aug-16 | Adult | Female |  |
| 277 | Sep-16 | Juvenile | Male |  |
| 278 | Sep-16 | Adult | Female |  |
| 279 | Sep-16 | Adult | Male |  |
| 280 | Oct-16 | Adult | Female |  |
| 281 | Nov-16 | Juvenile | Female |  |
| 282 | Nov-16 | Adult | Female |  |
| 283 | Nov-16 | Adult | Female |  |
| 284 | Nov-16 | Adult | Female |  |
| 285 | Nov-16 | Adult | Female |  |
| 286 | Nov-16 | Adult | Male |  |
| 287 | Dec-16 | Adult | Female |  |
| 288 | Dec-16 | Adult | Female |  |
| 289 | Dec-16 | Adult | Male |  |
| 290 | Dec-16 | Juvenile | Male |  |
| 291 | Dec-16 | Adult | Male |  |
| 292 | Jan-17 | Juvenile | Female |  |
| 293 | Jan-17 | Adult | Male |  |
| 294 | Jan-17 | Adult | Female |  |
| 295 | Jan-17 | Juvenile | Female |  |
| 296 | Feb-17 | Adult | Male |  |
| 297 | Feb-17 | Adult | Male |  |
| 298 | Nov-16 | Adult | Male |  |
| 299 | Jan-17 | Adult | Male |  |
| 300 | Jan-17 | Adult | Male |  |
| 301 | Jan-17 | Adult | Female |  |
| 302 | Jan-17 | Adult | Male |  |
| 303 | Feb-17 | Adult | Male |  |
| 304 | Jan-15 | Adult | Male |  |
| 305 | Jan-15 | Adult | Male |  |
| 306 | Mar-15 | Adult | Male |  |
| 307 | Jun-15 | Adult | Male |  |
| 308 | Jun-15 | Adult | Male |  |
| 309 | Aug-15 | Adult | Female |  |
| 310 | Aug-15 | Adult | Female |  |
| 311 | Aug-15 | Adult | Female |  |
| 312 | Aug-15 | Adult | Male |  |
| 313 | Aug-15 | Adult | Female |  |
| 314 | Aug-15 | Adult | Female |  |
| 315 | Aug-15 | Juvenile | Female |  |
| 316 | Aug-15 | Juvenile | Female |  |
| 317 | Aug-15 | Juvenile | Male |  |
| 318 | Aug-15 | Adult | Male |  |
| 319 | Aug-15 | Adult | Male |  |
| 320 | Aug-15 | Adult | Female |  |
| 321 | Aug-15 | Juvenile | Male |  |
| 322 | Aug-15 | Juvenile | Male |  |
| 323 | Aug-15 | Adult | Female |  |
| 324 | Sep-15 | Adult | Male |  |
| 325 | Sep-15 | Adult | Female |  |
| 326 | Oct-15 | Adult | Male |  |
| 327 | Oct-15 | Adult | Female |  |
| 328 | Oct-15 | Adult | Male |  |
| 329 | Oct-15 | Adult | Female |  |
| 330 | Oct-15 | Juvenile | Male |  |
| 331 | Oct-15 | Adult | Male |  |
| 332 | Dec-16 | Adult | Female |  |
| 333 | Dec-16 | Adult | Female |  |
| 334 | Dec-16 | Juvenile | Female |  |
| 335 | Dec-16 | Adult | Female |  |
| 336 | Dec-16 | Juvenile | Male |  |
| 337 | Dec-16 | Adult | Female |  |
| 338 | Jan-17 | Adult | Female |  |
| 339 | Feb-17 | Juvenile | Male |  |
| 340 | Feb-17 | Adult | Female |  |
| 341 | Feb-17 | Adult | Female |  |
| 342 | Feb-17 | Juvenile | Male |  |
| 343 | Mar-17 | Adult | Male |  |
| 344 | Mar-17 | Adult | Male |  |
| 345 | Mar-17 | Adult | Female |  |
| 346 | Mar-17 | Adult | Male |  |
| 347 | Mar-17 | Adult | Female |  |
| 348 | Aug-15 | Adult | Female |  |
| 349 | Nov-15 | Adult | Male |  |
| 350 | Feb-16 | Adult | Female |  |
| 351 | May-16 | Adult | Male |  |
| 352 | Aug-16 | Adult | Male |  |
| 353 | Sep-16 | Juvenile | Male |  |
| 354 | Sep-16 | Juvenile | Male |  |
| 355 | Sep-16 | Adult | Male |  |
| 356 | Nov-16 | Juvenile | Male |  |
| 357 | Nov-16 | Adult | Male |  |
| 358 | Dec-16 | Adult | Female |  |
| 359 | Dec-16 | Adult | Male |  |
| 360 | Jan-17 | Juvenile | Male |  |
| 361 | Sep-15 | Adult | Male |  |
| 362 | Jul-15 | Adult | Female |  |
| 363 | Aug-15 | Adult | Male |  |
| 364 | Oct-15 | Adult | Male |  |
| 365 | Oct-10 | Adult | Male |  |
| 366 | Oct-10 | Adult | Male |  |
| 367 | Nov-10 | Adult | Male |  |
| 368 | Nov-10 | Adult | Male |  |
| 369 | Nov-10 | Adult | Female |  |
| 370 | Mar-16 | Adult | Female |  |
| 371 | Mar-16 | Adult | Female |  |
| 372 | May-16 | Adult | Male |  |
| 373 | May-16 | Adult | Male |  |
| 374 | May-16 | Adult | Male |  |
| 375 | May-16 | Adult | Female |  |
| 376 | May-16 | Adult | Male |  |
| 377 | May-16 | Adult | Male |  |
| 378 | Jul-16 | Adult | Female |  |
| 379 | Aug-16 | Adult | Male |  |
| 380 | Aug-16 | Adult | Female |  |
| 381 | Aug-16 | Adult | Male |  |
| 382 | Aug-16 | Adult | Female |  |
| 383 | Aug-16 | Adult | Female |  |
| 384 | Aug-16 | Adult | Female |  |
| 385 | Aug-16 | Adult | Male |  |
| 386 | Aug-16 | Adult | Female |  |
| 387 | Aug-16 | Adult | Female |  |
| 388 | Aug-16 | Adult | Male |  |
| 389 | Aug-16 | Adult | Male |  |
| 390 | Aug-16 | Adult | Female |  |
| 391 | Aug-16 | Adult | Male |  |
| 392 | Aug-16 | Adult | Male |  |
| 393 | Aug-16 | Adult | Male |  |
| 394 | Sep-16 | Adult | Male |  |
| 395 | Sep-16 | Adult | Female |  |
| 396 | Sep-16 | Adult | Female |  |
| 397 | Nov-16 | Adult | Male |  |
| 398 | Nov-16 | Adult | Female |  |
| 399 | Nov-16 | Adult | Male |  |
| 400 | Dec-16 | Juvenile | Male |  |
| 401 | Dec-16 | Adult | Male |  |
| 402 | Dec-16 | Adult | Female |  |
| 403 | Nov-16 | Adult | Female |  |
| 404 | Dec-16 | Adult | Female |  |
| 405 | Jan-17 | Juvenile | Female |  |
| 406 | Feb-17 | Juvenile | Male |  |
| 407 | Aug-16 | Adult | Male |  |
| 408 | Mar-16 | Adult | Male | 20 |
| 409 | Aug-15 | Adult | Male |  |
| 410 | Aug-15 | Adult | Male |  |
| 411 | Aug-15 | Adult | Female |  |
| 412 | Aug-15 | Adult | Female |  |
| 413 | Aug-15 | Adult | Female |  |
| 414 | Aug-15 | Adult | Male |  |
| 415 | Aug-15 | Juvenile | Female |  |
| 416 | Nov-15 | Adult | Female |  |
| 417 | Dec-16 | Juvenile | Male |  |
| 418 | Dec-16 | Juvenile | Male |  |
| 419 | Dec-16 | Juvenile | Male |  |
| 420 | Feb-10 | Adult | Male |  |
| 421 | Mar-10 | Adult | Male |  |
| 422 | Mar-10 | Adult | Male |  |
| 423 | Feb-11 | Adult | Male |  |
| 424 | Apr-11 | Adult | Male |  |
| 425 | Apr-11 | Juvenile | Female |  |
| 426 | Dec-16 | Adult | Female |  |
| 427 | Jan-17 | Adult | Male |  |
| 428 | May-16 | Adult | Female | 25 |
| 429 | Aug-16 | Adult | Female |  |
| 430 | Aug-16 | Juvenile | Male |  |
| 431 | Sep-16 | Adult | Male |  |
| 432 | Jun-16 | Juvenile | Male |  |
| 433 | Oct-16 | Adult | Female |  |
| 434 | Nov-16 | Juvenile | Male |  |
| 435 | Nov-16 | Juvenile | Female |  |
| 436 | Dec-16 | Adult | Male |  |
| 437 | Dec-16 | Adult | Male |  |
| 438 | Oct-15 | Adult | Male |  |
| 439 | Jan-16 | Juvenile | Female |  |
| 440 | Jan-16 | Juvenile | Female |  |
| 441 | Jan-16 | Adult | Female |  |
| 442 | Jan-16 | Juvenile | Male |  |
| 443 | Mar-16 | Juvenile | Male |  |
| 444 | Mar-16 | Juvenile | Male |  |
| 445 | Mar-16 | Adult | Male |  |
| 446 | Aug-16 | Juvenile | Male |  |
| 447 | Aug-16 | Juvenile | Male |  |
| 448 | Nov-16 | Adult | Male |  |
| 449 | Nov-16 | Adult | Male |  |
| 450 | Nov-16 | Adult | Female |  |
| 451 | Dec-16 | Adult | Male |  |
| 452 | Dec-16 | Adult | Female |  |
| 453 | Jan-17 | Adult | Male | 30 |
| 454 | Mar-16 | Adult | Female |  |
| 455 | Jun-15 | Juvenile | Male |  |
| 456 | Jun-15 | Juvenile | Male |  |
| 457 | Feb-16 | Juvenile | Male |  |
| 458 | Feb-16 | Juvenile | Male |  |
| 459 | May-16 | Adult | Female |  |
| 460 | Mar-11 | Juvenile | Female |  |
| 461 | Apr-11 | Adult | Female |  |
| 462 | Apr-11 | Adult | Female |  |
| 463 | Apr-11 | Adult | Female |  |
| 464 | Apr-11 | Juvenile | Female |  |
| 465 | Apr-11 | Adult | Male |  |
| 466 | Apr-11 | Adult | Male |  |
| 467 | Apr-11 | Adult | Male |  |
| 468 | Mar-16 | Adult | Female |  |
| 469 | Mar-16 | Juvenile | Female |  |
| 470 | May-16 | Juvenile | Female |  |
| 471 | May-16 | Juvenile | Female |  |
| 472 | Jan-16 | Adult | Female |  |
| 473 | Jan-17 | Adult | Male |  |
| 474 | Jan-17 | Adult | Male |  |
| 475 | Jan-17 | Adult | Female |  |
| 476 | Jan-17 | Juvenile | Male |  |
| 477 | Mar-16 | Adult | Female |  |
| 478 | Feb-17 | Juvenile | Male |  |
| 479 | May-10 | Juvenile | Male |  |
| 480 | May-10 | Juvenile | Male |  |
| 481 | Mar-10 | Juvenile | Male |  |
| 482 | May-16 | Adult | Female |  |
| 483 | Sep-16 | Adult | Female | 12 |
| 484 | Sep-16 | Adult | Female |  |
| 485 | Sep-16 | Adult | Female |  |
| 486 | Sep-16 | Adult | Male |  |
| 487 | Sep-16 | Adult | Female |  |
| 488 | Sep-16 | Adult | Male |  |
| 489 | Nov-16 | Juvenile | Female |  |
| 490 | Nov-16 | Juvenile | Male |  |
| 491 | Nov-16 | Adult | Male |  |
| 492 | Dec-16 | Adult | Female |  |
| 493 | Dec-16 | Adult | Male |  |
| 494 | Jan-17 | Adult | Female |  |
| 495 | Oct-15 | Adult | Female | 90 |
| 496 | Oct-15 | Adult | Female |  |
| 497 | Oct-15 | Adult | Female |  |
| 498 | Feb-16 | Adult | Female |  |
| 499 | Mar-16 | Adult | Female |  |
| 500 | Apr-16 | Adult | Female |  |
| 501 | Sep-16 | Adult | Female |  |
| 502 | Sep-16 | Adult | Female |  |
| 503 | Dec-16 | Adult | Female |  |
| 504 | Dec-16 | Adult | Male |  |
| 505 | Nov-16 | Juvenile | Male |  |
| 506 | Jul-15 | Adult | Female |  |
| 507 | Aug-15 | Adult | Male |  |
| 508 | Aug-15 | Adult | Male |  |
| 509 | Oct-15 | Adult | Male |  |
| 510 | Nov-15 | Adult | Male |  |
| 511 | Jun-16 | Adult | Male |  |
| 512 | Aug-16 | Adult | Male |  |
| 513 | Nov-16 | Juvenile | Male |  |
| 514 | Dec-16 | Adult | Female |  |
| 515 | Dec-16 | Adult | Female |  |
| 516 | Feb-17 | Adult | Female |  |
| 517 | Sep-15 | Adult | Female |  |
| 518 | Mar-17 | Adult | Male |  |
| 519 | Mar-17 | Adult | Female |  |
| 520 | Jun-15 | Adult | Male |  |
| 521 | Jun-15 | Adult | Male |  |
| 522 | Jun-15 | Juvenile | Male |  |
| 523 | Jul-15 | Juvenile | Male |  |
| 524 | Jul-15 | Juvenile | Male |  |
| 525 | Sep-15 | Juvenile | Female |  |
| 526 | Oct-15 | Juvenile | Female |  |
| 527 | Oct-15 | Adult | Male |  |
| 528 | Oct-15 | Adult | Male |  |
| 529 | Oct-15 | Adult | Male |  |
| 530 | Nov-15 | Adult | Male |  |
| 531 | Nov-15 | Adult | Female |  |
| 532 | Feb-16 | Adult | Female |  |
| 533 | Mar-16 | Juvenile | Female |  |
| 534 | May-16 | Adult | Male |  |
| 535 | May-16 | Adult | Female |  |
| 536 | Jun-16 | Adult | Female |  |
| 537 | Jul-16 | Adult | Female |  |
| 538 | Aug-16 | Adult | Male |  |
| 539 | Aug-16 | Adult | Female |  |
| 540 | Aug-16 | Adult | Female |  |
| 541 | Aug-16 | Adult | Female |  |
| 542 | Sep-16 | Adult | Male |  |
| 543 | Oct-16 | Adult | Female |  |
| 544 | Nov-16 | Adult | Female |  |
| 545 | Dec-16 | Adult | Female |  |
| 546 | Dec-16 | Adult | Female |  |
| 547 | Jan-17 | Adult | Female |  |
| 548 | Jan-17 | Adult | Female |  |
| 549 | Jun-15 | Adult | Female |  |
| 550 | Jul-15 | Juvenile | Male |  |
| 551 | Sep-15 | Adult | Male |  |
| 552 | Sep-15 | Adult | Male |  |
| 553 | Oct-15 | Adult | Female |  |
| 554 | Oct-15 | Adult | Male |  |
| 555 | Oct-15 | Adult | Female |  |
| 556 | Oct-15 | Adult | Male |  |
| 557 | Oct-15 | Adult | Female |  |
| 558 | Oct-15 | Adult | Male |  |
| 559 | Oct-15 | Adult | Female |  |
| 560 | Oct-15 | Adult | Male |  |
| 561 | Oct-15 | Adult | Male |  |
| 562 | Oct-15 | Adult | Female |  |
| 563 | Oct-15 | Adult | Male |  |
| 564 | Oct-15 | Adult | Male |  |
| 565 | Oct-15 | Juvenile | Male |  |
| 566 | Oct-15 | Adult | Male |  |
| 567 | Oct-15 | Adult | Male |  |
| 568 | Nov-15 | Adult | Female |  |
| 569 | Nov-15 | Adult | Female |  |
| 570 | Jan-16 | Adult | Male |  |
| 571 | Mar-16 | Adult | Female |  |
| 572 | Apr-16 | Adult | Female |  |
| 573 | May-16 | Juvenile | Male |  |
| 574 | May-16 | Adult | Female |  |
| 575 | Sep-16 | Adult | Male |  |
| 576 | Nov-16 | Adult | Female |  |
| 577 | Nov-16 | Adult | Female |  |
| 578 | Nov-16 | Adult | Male |  |
| 579 | Nov-16 | Adult | Male |  |
| 580 | Dec-16 | Adult | Male |  |
| 581 | Dec-16 | Adult | Female |  |
| 582 | Dec-16 | Adult | Female |  |
| 583 | Dec-16 | Adult | Female |  |
| 584 | Dec-16 | Adult | Female |  |
| 585 | Dec-16 | Adult | Female |  |
| 586 | Jan-17 | Adult | Male |  |
| 587 | Jan-17 | Adult | Female |  |
| 588 | Mar-16 | Juvenile | Male |  |
| 589 | Jan-17 | Juvenile | Female | 19 |
| 590 | Jan-17 | Juvenile | Male |  |
| 591 | Jan-17 | Juvenile | Female |  |
| 592 | Jan-17 | Juvenile | Female |  |
| 593 | Feb-17 | Juvenile | Male |  |
| 594 | Feb-17 | Juvenile | Female |  |
| 595 | Feb-17 | Adult | Female |  |
| 596 | Feb-17 | Juvenile | Male |  |
| 597 | Feb-17 | Adult | Female |  |
| 598 | Mar-17 | Adult | Female |  |
| 599 | Mar-17 | Adult | Male |  |
| 600 | Mar-17 | Adult | Female |  |
| 601 | Mar-17 | Adult | Female |  |
| 602 | Mar-17 | Juvenile | Male |  |
| 603 | Mar-17 | Juvenile | Female |  |
| 604 | Mar-17 | Adult | Female |  |
| 605 | Mar-17 | Adult | Female |  |
| 606 | Mar-17 | Adult | Female |  |
| 607 | Feb-16 | Adult | Female |  |
| 608 | Apr-16 | Adult | Male | 18 |
| 609 | Apr-16 | Adult | Male |  |
| 610 | May-16 | Adult | Male |  |
| 611 | Jul-16 | Adult | Male |  |
| 612 | Dec-16 | Juvenile | Female |  |
| 613 | Apr-10 | Adult | Male |  |
| 614 | Sep-15 | Adult | Female |  |
| 615 | Nov-15 | Adult | Female |  |
| 616 | Jul-15 | Juvenile | Male |  |
| 617 | Jul-15 | Adult | Male |  |
| 618 | May-16 | Adult | Male |  |
| 619 | Jul-15 | Juvenile | Female |  |
| 620 | May-16 | Adult | Female |  |
| 621 | May-16 | Adult | Male |  |
| 622 | May-16 | Juvenile | Male |  |
| 623 | Mar-16 | Adult | Female |  |
| 624 | Jan-16 | Adult | Female |  |
| 625 | Jan-16 | Adult | Female |  |
| 626 | Mar-17 | Adult | Male | 7 |
| 627 | Jul-15 | Adult | Female |  |
| 628 | Nov-15 | Juvenile | Female |  |
| 629 | Jan-16 | Adult | Female |  |
| 630 | Feb-16 | Adult | Female |  |
| 631 | Feb-16 | Juvenile | Male |  |
| 632 | Feb-16 | Adult | Female |  |
| 633 | Sep-16 | Adult | Male | 5 |
| 634 | Sep-16 | Adult | Female |  |
| 635 | Dec-16 | Juvenile | Female |  |
| 636 | Apr-15 | Adult | Female |  |
| 637 | Sep-15 | Adult | Male |  |
| 638 | Apr-10 | Adult | Male | 5 |
| 639 | Apr-10 | Juvenile | Male |  |
| 640 | May-10 | Adult | Male |  |
| 641 | May-10 | Adult | Male |  |
| 642 | Nov-10 | Adult | Female |  |
| 643 | Mar-10 | Juvenile | Male | 3 |
| 644 | Mar-10 | Juvenile | Male |  |
| 645 | Mar-10 | Juvenile | Female |  |
| 646 | Feb-10 | Adult | Male | 2 |
| 647 | Feb-10 | Adult | Male |  |
| 648 | Jan-16 | Adult | Male | 2 |
| 649 | Feb-16 | Adult | Male |  |
| 650 | Feb-10 | Adult | Male | 1 |
| 651 | Aug-15 | Adult | Male | 1 |
| 652 | Feb-17 | Adult | Male | 1 |
| 653 | Jul-15 | Adult | Female | 1 |
| 654 | Apr-10 | Juvenile | Male | 1 |
| 655 | Mar-17 | Adult | Male | 1 |
| 656 | Apr-11 | Adult | Male | 1 |
| 657 | Dec-16 | Adult | Male | 1 |
| 658 | Apr-11 | Adult | Male | 1 |
| 659 | Jul-16 | Adult | Male | 1 |
| 660 | Dec-16 | Juvenile | Male | 1 |
