# Supplementary material for: Prevalence of simian malaria parasites in macaques of Singapore
Source: PLoS Negl Trop Dis. 2021 Jan 25;15(1):e0009110. doi: 10.1371/journal.pntd.0009110 (PMC7861519; doi:10.1371/journal.pntd.0009110)
Supplement: S3 Table — (DOCX) [file pntd.0009110.s003.docx]

S3 Table: Summary of positive controls used in the study.

*Plasmodium falciparum*, *P. vivax, P. ovale* and *P. knowlesi* were obtained through the routine malaria diagnostic blood samples received by the Environmental Health Institute (EHI). *Plasmodium coatneyi, P. cynolmogi, P. fieldi and P.inui* genomic DNA were extracted from blood spots obtained from the Centre for Disease Control and Prevention, USA.

| Species | Source | Date of bloodspot |
| --- | --- | --- |
| *Plasmodium coatneyi* | Isolated from *Anopheles hackeri* followed by propagation in rhesus monkey | 22/02/2010 |
| *Plasmodium cynomolgi* | Propagated in rhesus monkey | 12/04/2001 |
| *Plasmodium fieldi* | Isolated from *Anopheles hackeri* followed by propagation in rhesus monkey | 24/04/2006 |
| *Plasmodium inui* | Isolated from leaf-bandedmonkey | 23/03/2004 |
